# Supplementary material for: Building of a new Spectra for the identification of Phytobacter spp., an emerging Enterobacterales, using MALDI Biotyper
Source: Microbiol Spectr. 2024 Sep 9;12(10):e01107-24. doi: 10.1128/spectrum.01107-24 (PMC11448068; doi:10.1128/spectrum.01107-24)
Supplement: Supplemental material — Tables S1 and S2. [file spectrum.01107-24-s0001.docx]

**Supplemental Table 1: Results for MALDI-TOF-MS (MALDI Biotyper® Bruker) before the new *Phytobacter* spp library creation.**

| **SAMPLE AND FINAL WGS ID** | **PRE NEW LIBRARY** | **ORGANISM (BEST MATCH)** | **SCORE MIN (VALUE)** | **SCORE MAX (VALUE)** | **AVERAGE** | **ORGANISM**  **(SECOND-BEST VALUE)** | **SCORE MIN (VALUE)** | **SCORE MAX (VALUE)** | **AVERAGE** |
| --- | --- | --- | --- | --- | --- | --- | --- | --- | --- |
| **5108RM**  *P. diazotrophicus* | Matrix | Test 1 - *Enterobacter cloacae*  Test 2 - *Escherichia coli* | 1.73  1.64 | 1.73  1.64 | 1.73  1.64 | Test 1 -*Escherichia hermannii*  Test 2 - *Escherichia coli* | 1.71  1.60 | 1.71  1.60 | 1.71  1.60 |
|  | Formic acid | Test 1 – *Enterobacter kobei*  Test 2 – *Enterobacter cloacae* | 1.72  1.78 | 1.72  1.78 | 1.72  1.78 | Test 1 - *Escherichia coli*  Test 2 - *Escherichia coli* | 1.68  1.69 | 1.68  1.69 | 1.68  1.69 |
| **5110RM**  *P. diazotrophicus* | Matrix | Test 1 – *Enterobacter asburiae*  Test 2 – *Escherichia hermannii* | 1.64  1.50 | 1.64  1.50 | 1.64  1.50 | Test 1 - *Klebsiella oxytoca*  Test 2 - *Hafnia alvei* | 1.62  1.47 | 1.62  1.47 | 1.62  1.47 |
|  | Formic acid | Test 1 - NO IDP  Test 2 – *Escherichia coli* | -  1.79 | -  1.79 | -  1.79 | Test 1 - NO IDP  Test 2 – *Escherichia hermannii* | -  1.70 | -  1.70 | -  1.70 |
| **26929RM**  *P. diazotrophicus* | Matrix | ***Phytobacter ursingi****i* | 1.81 | 2.14 | 1.93 | *Klebsiella oxytoca* | 1.79 | 1.79 | 1.79 |
| **28192RM**  *P. ursingii* | Matrix | ***Phytobacter ursingii*** | 1.82 | 2.16 | 2.03 | Test 1 - *Enterobacter cloacae*  Test 2 - *Cronobacter* sp. | 1.76  1.79 | 1.86  1.79 | 1.80  1.79 |
| **29310RM**  *P. diazotrophicus* | Matrix | ***Phytobacter ursingii*** | 1.78 | 1.98 | 1.88 | *Raoultella ornithinolytica* | 1.82 | 1.84 | 1.83 |
| **32062RM**  *P. ursingii* | Matrix | ***Phytobacter ursingii*** | 1.78 | 2.06 | 1.94 | *Cronobacter* sp | 1.81 | 1.81 | 1.81 |
| **32064RM**  *P. diazotrophicus* | Matrix | ***Phytobacter ursingii*** | 1.90 | 2.23 | 2.03 | Test 1 - *Raoultella ornithinolytica*  Test 2 - *Pseudescherichia vulneris* | 1.84  1.79 | 1.88  1.83 | 1.86  1.81 |
| **32066RM**  *P. diazotrophicus* | Matrix | Test 1 – *Cronobacter sp.*  Test 2 – *Escherichia coli* | 1.72  1.94 | 1.72  1.94 | 1.72  1.94 | Test 1 – *Enterobacter kobei*  Test 2 - NO IDP | 1.89  - | 1.89  - | 1.89  - |
|  | Formic acid | Test 1 – *Salmonella sp*.  Test 2 – *Klebsiella oxytoca* | 1.77  1.94 | 1.77  1.94 | 1.77  1.94 | Test 1 – *Enterobacter kobei*  Test 2 – ***Phytobacter ursingii*** | 1.85  1.76 | 1.85  1.76 | 1.85  1.76 |
| **32366RM**  *P. diazotrophicus* | Matrix | Test 1 – *Cronobacter sp.*  Test 2 – *Escherichia coli* | 1.94  1.72 | 1.94  1.72 | 1.94  1.72 | Test 1 – *Enterobacter kobei*  Test 2 - ***Phytobacter ursingii*** | 1.89  1.69 | 1.89  1.69 | 1.89  1.69 |
|  | Formic acid | Test 1 – *Escherichia coli*  Test 2 – *Klebsiella oxytoca* | 1.77  1.86 | 1.77  1.86 | 1.77  1.86 | Test 1 – *Escherichia hermannii*  Test 2 – ***Phytobacter ursingii*** | 1.76  1.83 | 1.76  1.83 | 1.76  1.83 |
| **33822RM**  *P. diazotrophicus* | Matrix | Test 1 – *Escherichia coli*  Test 2 – *Citrobacter farmeri* | 1.79  1.78 | 1.79  1.78 | 1.79  1.78 | Test 1 – *Pseudescherichia vulneris*  Test 2 – *Escherichia coli* | 1.77  1.70 | 1.77  1.70 | 1.77  1.70 |
|  | Formic acid | Test 1 – ***Phytobacter ursingii***  Test 2 – *Escherichia coli* | 2.08  1.96 | 2.08  1.96 | 2.08  1.96 | Test 1 –*Cronobacter sp.*  Test 2 – *Escherichia coli* | 1.75  1.88 | 1.75  1.88 | 1.75  1.88 |
| **38394RM**  *P. diazotrophicus* | Matrix | Test 1 *– Enterobacter hormaechei*  Test 2 – *Pluralibacter gergoviae* | 1.81  1.80 | 1.81  1.80 | 1.81  1.80 | Test 1 – ***Phytobacter ursingii***  Test 2 – *Enterobacter kobei* | 1.78  1.76 | 1.78  1.76 | 1.78  1.76 |
|  | Formic acid | Test 1 –*Cronobacter sp*  Test 2 – ***Phytobacter ursingii*** | 1.90  2.13 | 1.90  2.13 | 1.90  2.13 | Test 1 *– Salmonella sp*  Test 2 – *Klebsiella aerogenes* | 1.78  1.91 | 1.78  1.91 | 1.78  1.91 |
| **38397RM**  *P. diazotrophicus* | Matrix | Test 1 – ***Phytobacter ursingii***  Test 2 – ***Phytobacter ursingii*** | 1.96  1.91 | 1.96  1.91 | 1.96  1.91 | Test 1 – *Klebsiella oxytoca*  Test 2 – *Escherichia coli* | 1.95  1.81 | 1.95  1.81 | 1.95  1.81 |
|  | Formic acid | Test 1 – *Raoultella ornithinolytica*  Test 2 – ***Phytobacter ursingii*** | 1.78  2.19 | 1.78  2.19 | 1.78  2.19 | Test 1 –*Enterobacter cloacae*  Test 2 – *Enterobacter kobei* | 1.75  1.87 | 1.75  1.87 | 1.75  1.87 |
| **38453RM**  *P. diazotrophicus* | Matrix | Test 1 – *Enterobacter kobei*  Test 2 – ***Phytobacter ursingii*** | 1.86  1.97 | 1.86  1.97 | 1.86  1.97 | Test 1 – ***Phytobacter ursingii***  Test 2 – *Pluralibacter gergoviae* | 1.81  1.80 | 1.81  1.80 | 1.81  1.80 |
|  | Formic acid | Test 1 – ***Phytobacter ursingii***  Test 2 – *Raoultella ornithinolytica* | 2.14  1.73 | 2.14  1.73 | 2.14  1.73 | Test 1 – *Pluralibacter gergoviae*  Test 2 – *Cronobacter* *sp.* | 1.95  1.72 | 1.95  1.72 | 1.95  1.72 |
| **38468RM**  *P. diazotrophicus* | Matrix | Test 1 – *Escherichia coli*  Test 2 – *Klebsiella oxytoca* | 1.87  1.89 | 1.87  1.89 | 1.87  1.89 | Test 1 – *Enterobacter asburiae*  Test 2 – *Enterobacter cloacae* | 1.85  1.82 | 1.85  1.82 | 1.85  1.82 |
|  | Formic acid | Test 1 – *Enterobacter cloacae*  Test 2 – ***Phytobacter ursingii*** | 1.92  2.13 | 1.92  2.13 | 1.92  2.13 | Test 1 – *Pseudescherichia vulneris*  Test 2 –*Klebsiella oxytoca* | 1.89  1.86 | 1.89  1.86 | 1.89  1.86 |
| **34630RM**  *P. ursingii* | Matrix | Test 1 – *Escherichia coli*  Test 2 - *Escherichia coli* | 1.91  1.67 | 1.91  1.67 | 1.91  1.67 | Test 1 – *Klebsiella oxytoca*  Test 2 - *Klebsiella oxytoca* | 1.85  1.65 | 1.85  1.65 | 1.85  1.65 |
|  | Formic acid | Test 1 – *Citrobacter koseri*  Test 2 – *Escherichia coli* | 1.78  1.91 | 1.78  1.91 | 1.78  1.91 | Test 1 – *Enterobacter cloacae*  Test 2 – *Raoultella ornithinolytica* | 1.76  1.81 | 1.76  1.81 | 1.76  1.81 |
| **37396RM**  *P. ursingii* | Matrix | Test 1 – *Escherichia coli*  Test 2 - *Enterobacter kobei* | 1.81  1.64 | 1.81  1.64 | 1.81  1.64 | Test 1 – *Klebsiella oxytoca*  Test 2 - *Enterobacter cloacae* | 1.79  1.60 | 1.79  1.60 | 1.79  1.60 |
|  | Formic acid | Test 1 – *Escherichia coli*  Test 2 – *Escherichia hermannii* | 1.78  1.75 | 1.78  1.75 | 1.78  1.75 | Test 1 – *Escherichia coli*  Test 2 – *Escherichia coli* | 1.70  1.65 | 1.70  1.65 | 1.70  1.65 |

**Supplemental Table 2: Results for MALDI-TOF-MS (MALDI Biotyper® Bruker) after creating and activating the new *Phytobacter* spp. library.**

| **SAMPLE AND FINAL WGS ID** | **POST NEW LIBRARY** | **ORGANISM (BEST MATCH)** | **SCORE MIN (VALUE)** | **SCORE MAX (VALUE)** | **AVERAGE** | **ORGANISM**  **(SECOND-BEST VALUE)** | **SCORE MIIN (VALUE)** | **SCORE MAX (VALUE)** | **AVERAGE** |
| --- | --- | --- | --- | --- | --- | --- | --- | --- | --- |
| **5108RM**  *P. diazotrophicus* | Matrix | Test 1 – NPF  Test 2 - NRI | - | - | - | Test 1 – NPF  Test 2 - NRI | - | - | - |
|  | Formic acid | ***Phytobacter diazotrophicus*** | 1.63 | 1.65 | 1.64 | *Phytobacter ursingii* | 1.16 | 1.73 | 1.44 |
| **5110RM**  *P. diazotrophicus* | Matrix | Test 1 – NPF  Test 2 - NRI | - | - | - | Test 1 – NPF  Test 2 - NRI | - | - | - |
|  | Formic acid | ***Phytobacter diazotrophicus*** | 2.22 | 2.25 | 2.23 | *Phytobacter ursingii* | 1.93 | 2.09 | 2.01 |
| **32066RM**  *P. diazotrophicus* | Matrix | ***Phytobacter diazotrophicus*** |  |  |  | *Phytobacter ursingii* | 1.87 | 1.92 | 1.90 |
|  | Formic acid | ***Phytobacter diazotrophicus*** | 2.03 | 2.24 | 2.33 | *Phytobacter ursingii* | 1.94 | 2.07 | 2.26 |
| **32366RM**  *P. diazotrophicus* | Matrix | ***Phytobacter diazotrophicus*** | 2.01 | 2.02 | 2.01 | *Phytobacter ursingii* | 1.87 | 1.92 | 1.90 |
|  | Formic acid | ***Phytobacter diazotrophicus*** | 1.96 | 2.28 | 2.12 | *Phytobacter ursingii* | 1.61 | 2.26 | 1.93 |
| **33822RM**  *P. diazotrophicus* | Matrix | ***Phytobacter diazotrophicus*** | 1.63 | 1.70 | 1.66 | *Phytobacter ursingii* | 1.31 | 1.35 | 1.33 |
|  | Formic acid | ***Phytobacter diazotrophicus*** | 1.86 | 1.90 | 1.88 | *Phytobacter ursingii* | 1.85 | 2.08 | 1.96 |
| **38394RM**  *P. diazotrophicus* | Matrix | ***Phytobacter diazotrophicus*** | 1.26 | 1.46 | 1.36 | *Phytobacter ursingii* | 1,06 | 1.50 | 1.28 |
|  | Formic acid | ***Phytobacter diazotrophicus*** | 1.77 | 2.12 | 1.94 | *Phytobacter ursingii* | 1.61 | 2.22 | 1.91 |
| **38397RM**  *P. diazotrophicus* | Matrix | ***Phytobacter diazotrophicus*** | 1.36 | 1.37 | 1.36 | *Phytobacter ursingii* | 1.26 | 1.33 | 1.29 |
|  | Formic acid | ***Phytobacter diazotrophicus*** | 1.81 | 1.99 | 1.90 | *Phytobacter ursingii* | 1.77 | 2.20 | 1.98 |
| **38453RM**  *P. diazotrophicus* | Matrix | ***Phytobacter diazotrophicus*** | 1.94 | 1.97 | 1.95 | *Phytobacter ursingii* | 1.61 | 1.68 | 1.64 |
|  | Formic acid | ***Phytobacter diazotrophicus*** | 1.96 | 2.19 | 2.07 | *Phytobacter ursingii* | 1.85 | 2.04 | 1,94 |
| **38468RM**  *P. diazotrophicus* | Matrix | ***Phytobacter diazotrophicus*** | 1.67 | 1.89 | 1.78 | *Phytobacter ursingii* | 1.33 | 1.53 | 1.43 |
|  | Formic acid | *Phytobacter ursingii* | 2.07 | 2.23 | 2.15 | ***Phytobacter diazotrophicus*** | 1.97 | 2.16 | 2.06 |
| **34630RM**  *P. ursingii* | Matrix | *Phytobacter diazotrophicus* | 2 | 2.05 | 2.02 | ***Phytobacter ursingii*** | 1.95 | **2.01** | 1.98 |
|  | Formic acid | ***Phytobacter ursingii*** | 1.73 | **2.23** | 1.98 | *Phytobacter diazotrophicus* | 1.67 | 2.06 | 1.86 |
| **37396RM**  *P. ursingii* | Matrix | *Phytobacter diazotrophicus* | 1,21 | 1.71 | 1.46 | *Phytobacter ursingii* | 1,09 | 1.66 | 1.37 |
|  | Formic acid | ***Phytobacter ursingii*** | 2 | **2.15** | 2.07 | *Phytobacter diazotrophicus* | 1.82 | 1.96 | 1.89 |
| **ATCC 27981**  *P. diazotrophicus* | Matrix | *Phytobacter diazotrophicus* | 1.69 | 1.84 | 1.75 | *Phytobacter ursingii* | 1.78 | 1.79 | 1.78 |
|  | Formic acid | *Phytobacter ursingii* | 1.79 | 1.86 | 1.82 | *Phytobacter diazotrophicus* | 1,67 | 1.81 | 1.74 |
|  | Extraction | *Phytobacter ursingii* | 2.13 | 2.14 | 2.13 | ***Phytobacter diazotrophicus*** | 2.06 | **2.20** | 2.13 |
| **DSM17806 ^T^**  *P. diazotrophicus* | Matrix | *Phytobacter diazotrophicus* | 1.90 | 1.91 | 1.90 | *Phytobacter ursingii* | 1.84 | 1.86 | 1.85 |
|  | Formic acid | *Phytobacter diazotrophicus* | 1.89 | 2 | 1.94 | *Phytobacter ursingii* | 1.72 | 1.78 | 1.75 |
|  | Extraction | ***Phytobacter diazotrophicus*** | 2.03 | 2.27 | 2.15 | *Phytobacter ursingii* | 1.99 | 2.01 | 2.00 |

**Caption:** All 12 isolates were tested in triplicate with three methods: Matrix (Direct-TD); Formic Acid (Indirect-TE), and Extraction (Acetonitrile-EXT). Since the method with extraction, in most samples, had no peaks, these results were suppressed from the table except when ID was achieved.

NO IDP – No Identification Possible NRI- Not Reliable Identification NPF – No Peak Found WGS - Whole Genome Sequencing

Color coding - Colum 1 - Right species identified, with at least one score > 2.00; Right genera identified, with at least one score > 2.00; Right genera OR right species identified, with score > 1.69 and (Sample and Final ID) < 2.00; Right genera identified but within the second-best score, between 1.70-1.99; Not the right species identified within the organism's best and second-best match
